# 148. Imipenem-Relebactam activity and genotypic characteristics of Carbapenem-resistant *Enterobacterales* and *Pseudomonas aeruginosa* Isolates from Latin American Infections - Study for Monitoring Antimicrobial Resistance Trends (SMART) 2017 – 2020

**DOI:** 10.1093/ofid/ofac492.226

**Published:** 2022-12-15

**Authors:** Gustavo Mizuno, Thales Polis, Jacqueline Ferrari, Jacqueline Pavia, Elisa Beirão, Ana C Gales, Felipe Tuon, Alexandre Alcantara, Paula M Batista

**Affiliations:** MSD Brazil, Sao Paulo, Brazil, Sao Paulo, Sao Paulo, Brazil; MSD Brazil, Sao Paulo, Brazil, Sao Paulo, Sao Paulo, Brazil; MSD Brazil, Sao Paulo, Brazil, Sao Paulo, Sao Paulo, Brazil; MSD Colombia, Bogota, Colombia, Bogota, Distrito Capital de Bogota, Colombia; HOSPITAL MANDAQUI, SAO PAULO, Sao Paulo, Brazil; UNIFESP, Brazil; Laboratory of Emerging Infectious Diseases, Pontifícia Universidade Catolica do Paraná, CURITIBA, Parana, Brazil; MSD BRAZIL, SAO PAULO, Sao Paulo, Brazil; MSD BRAZIL, SAO PAULO, Sao Paulo, Brazil

## Abstract

**Background:**

Imipenem/relebactam (IMI/REL) is a combination of Imipenem with Relebactam, an inhibitor of class A and C β-lactamases and has been approved in the US and EU, but not in Latin America.

This report evaluates the in vitro activity of IMI/REL and comparators against Latin America (LATAM) *Enterobacterales* and *P. aeruginosa* (PSA) and the frequency of carbapenemase encoding genes (CEG) among gram-negative bacilli (GNB) isolated through the SMART Program (2017-2020).

**Methods:**

21606 nonconsecutive GNB isolates were collected in 10 LATAM countries. MICs for amikacin (AK), ceftazidime-avibactam (C-A), ceftolozane/tazobactam (C/T) and IMI/REL were determined by broth microdilution and interpreted by CLSI; a subset of *Enterobacterales* and *P. aeruginosa* carbapenem resistant was selected for characterization of carbapenemase encoding genes by PCR followed by DNA sequencing.

**Results:**

*Escherichia coli* (N=9872; EC) tested susceptible to > 96% of all antibiotics analyzed; for *P. aeruginosa* (N=4528), C/T and C-A had the best susceptibility rates (85.7 and 86.6% respectively); for *Enterobacter cloacae* (N=1091; ECL) and *Klebsiella pneumoniae* (N=6115; KPN), we note that only IMI/REL and C-A were ≥ 95%.

Profile of 2845 carbapenem resistant isolates was analysed and the main isolated agent in most countries was KPN, corresponding to 55% (1470), except in Mexico, Panama and Venezuela where PSA was the main carbapenemase producer.

The *bla*_kpc-2, 3_ were found in KPN 76.7, 79.2, 53.3, 52.1 and 85.3% in Argentina, Brazil, Colombia, Ecuador and Puerto Rico, respectively; Guatemala, Mexico, Venezuela presented *bla*_NDM-1_ in 74.3, 44.1 and 51.4%.

Among ECL *bla*_kpc-2_ (35.7%-Brazil, 48.2%-Colombia) and *bla*_NDM-1_(45.7%-Mexico) were most frequent, *bla*_VIM-24_ (16.6%) and *bla*_IMP-18_ (38.8%) were observed in Venezuela and Puerto Rico and in the latter, we observed first time reported *bla*_kpc-45_ in 27.7 %.

PSA expressed *bla*_kpc-2_ (33.33%- Colombia), Chile and Venezuela *bla*_VIM-2_ (44.3%, 58.8%); *bla*_spm-1_ occurred only in Brazil (6.9%).

Most common carbapenemase-producing bacteria in LATAM (n=2845)

Antimicrobial susceptibility of Enterobacterales and P. aeruginosa (full data analysis=21606)

**Figure:**
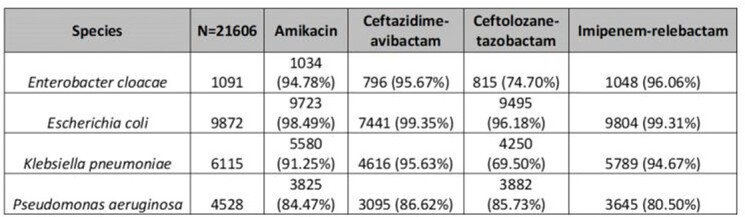

Most Frequent CEG detected among carbapenemase producing isolates in Latin American countries (Genotypical sample analyzed n=2845)

**Figure:**
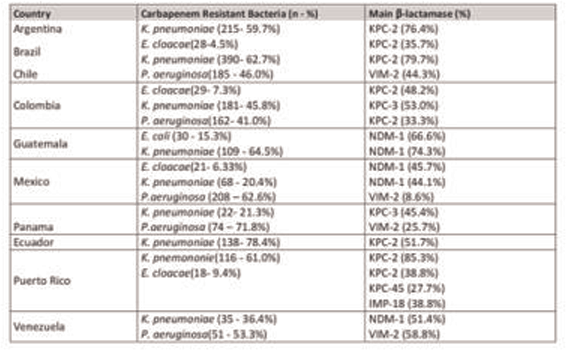

**Conclusion:**

The frequency of CEG is a threat in LATAM, mostly in *Enterobacterales*, whereas PSA as expected, has a lower frequency, but still a concern.

In LATAM, IMI/REL has shown relevant activity against CEG producers, showing it is an option for treatment infections caused by MDR strains.

**Disclosures:**

**Gustavo Mizuno, PharmD**, MSD Brazil: Employee **Thales Polis, MD**, MSD Brazil: Employee **Jacqueline Ferrari, MD**, MSD Brazil: Employee **Jacqueline Pavia, MD**, MSD COLOMBIA: EMPLOYEE **Elisa Beirão, MD**, MSD BRAZIL: Grant/Research Support **Ana C. Gales, MD**, MSD BRAZIL: Grant/Research Support **Felipe Tuon, MD**, MSD BRAZIL: Grant/Research Support **Alexandre Alcantara, PharmD**, MSD BRAZIL: EMPLOYEE **Paula M. Batista, PharmD**, MSD BRAZIL: EMPLOYEE.

